# Self-sampling is appropriate for detection of *Staphylococcus aureus*: a validation study

**DOI:** 10.1186/2047-2994-1-34

**Published:** 2012-11-08

**Authors:** Brigitte AGL van Cleef, Miranda van Rijen, Marianne Ferket, Jan AJW Kluytmans

**Affiliations:** 1 RIVM National Institute for Public Health and the Environment, Centre for Infectious Disease Control Netherlands, P.O. Box 1, 3720 BA, Bilthoven, the Netherlands; 2 St. Elisabeth hospital, Laboratory for Medical Microbiology and Immunology, Tilburg, the Netherlands; 3 Amphia Hospital Breda, Laboratory for Microbiology and Infection Control, Breda, the Netherlands; 4 VU University medical centre, Department of Medical Microbiology and Infection Prevention, Amsterdam, the Netherlands

**Keywords:** Self-sampling, *Staphylcococcus aureus*, MRSA, Validation

## Abstract

**Background:**

Studies frequently use nasal swabs to determine *Staphylococcus aureus* carriage. Self-sampling would be extremely useful in an outhospital research situation, but has not been studied in a healthy population. We studied the similarity of self-samples and investigator-samples in nares and pharynxes of healthy study subjects (hospital staff) in the Netherlands.

**Methods:**

One hundred and five nursing personnel members were sampled 4 times in random order after viewing an instruction paper: 1) nasal self-sample, 2) pharyngeal self-sample, 3) nasal investigator-sample, and 4) pharyngeal investigator-sample.

**Results:**

For nasal samples, agreement is 93% with a kappa coefficient of 0.85 (95% CI 0.74-0.96), indicating excellent agreement, for pharyngeal samples agreement is 83% and the kappa coefficient is 0.60 (95% CI 0.43-0.76), indicating good agreement. In both sampling sites self-samples even detected more *S. aureus* than investigator-samples.

**Conclusions:**

This means that self-samples are appropriate for detection of *Staphylococcus aureus* and methicillin-resistant *Staphylococcus aureus*.

## Background

*Staphylococcus aureus* (*S. aureus*) is a well-known human commensal on skin and mucous membranes, with as most important ecological niche the nares [1,2]. In addition to colonization, *S. aureus* can cause skin and soft tissue infections, and more severe infections like necrotizing pneumonia, osteomyelitis and sepsis, which are regularly seen in hospitals in patients with other comorbidities.

Studies frequently use nasal swabs to determine *S. aureus* carriage, sometimes supplemented by pharyngeal swabs to increase the validity of the sampling method [3-5]. Especially in research settings in populations outside hospitals, self-sampling is often performed to enhance response and lower travel costs and time for both the studied subject and the investigator [6,7].

The only study examining self-sampling for methicillin-resistant *S. aureus* (MRSA) is Lautenbach et al. [8]. They sampled 56 MRSA-positive inpatients in the US and compared different anatomic sites and patient-collected versus provider-collected samples. Agreement percentages of 82% (nose) and 91% (throat) were reported, demonstrating excellent agreement. However, the study population (US inpatients) is not comparable to European citizens considering health status and MRSA-prevalence; the latter is several times higher in the US compared to the Netherlands and Northern Europe [9,10]. Moreover, sampling order always was a sample collected by a provider, followed by a patient-collected sample. Lastly, instructions for self-sampling were not clear: were photographs available, was clearly stated which area to sample and with which technique?

We therefore studied the similarity of self-samples and investigator-samples in testing the presence of *S. aureus* in nares and pharynxes of healthy study subjects in the Netherlands, using random sample order and clear instructions.

## Methods

This crossover screening validation took place in April 2009. From nursing and technical personnel working in different wards in the Amphia hospital in Breda, the Netherlands, 4 samples were taken in random order: 1) nasal self-sample, 2) pharyngeal self-sample, 3) nasal investigator-sample, and 4) pharyngeal investigator-sample. A convenience sample size of around 100 subjects (400 samples) was determined.

Before sampling, a one page instruction was shown with photographs of both samplings (see Additional file 1), no spoken instructions were given in order to represent a true self-sample. The subject was advised to sample both inner nares, especially in the tip of the nose, and both tonsils or tonsillar arches with a different swab, both in a turning movement. Venturi Transystem swabs with Stuart medium were used (Copan Innovation, Italy). Before or after these two self-samples, the investigator sampled the nares and pharynx using the same technique as described above.

Swabs were inoculated on SA ID agar plates (bioMérieux, La Balme Les Grottes, France) and Columbia blood agar plates with 5% sheep blood, and enriched in Mueller-Hinton broth containing 6.5% NaCl. From the overnight Mueller-Hinton broth, 10 μl was streaked onto an SA ID agar plate. The results were read after overnight incubation at 35°C. Colonies showing green coloration were considered indicative for *S. aureus*, and were confirmed by standard techniques: colony morphology, coagulase slide test, DNase test, and a coagulase tube when discrepancies arose. Colonies with colours other than green, or no growth at all were considered negative. The procedure was performed as recommended by the manufacturer.

From 2x2 tables, percentage agreement was calculated. Cohen’s simple kappa coefficients with 95% Confidence Intervals (CI) were calculated with SAS, version 9.3 (SAS Institute Inc., Cary, NC, USA). A kappa coefficient of >0.6 was considered to indicate good agreement, a coefficient of >0.8 was considered to indicate excellent agreement, and a coefficient of 1 indicated perfect agreement. Gold standards were created combining self-samples and investigator-samples per sampling site; if either one was positive, that person was considered *S. aureus* positive on that site. Sensitivities were calculated with OpenEpi [11].

## Results

A total of 105 nursing and technical personnel members were sampled. Thirty percent (31/105) of nasal investigator-samples were *S. aureus* positive, compared to 34% (36/105) of self-samples. For pharyngeal samples, these numbers were 27% (28/105) and 34% (36/105), respectively. Table 1 shows that nasal self-samples and investigator-samples have an agreement of 93% and a kappa coefficient of 0.85 (CI 0.74-0.96). For pharyngeal samples an agreement of 83% and a kappa coefficient of 0.60 (CI 0.43-0.76) was obtained. In both sampling sites self-samples even dectected more *S. aureus* than investigator-samples.

**Table 1:** Self-samples versus investigator-samples per sampling site

**Nasal samples**		**Investigator-samples**		**Total**
		**SApos**	**SAneg**	
**Self-samples**	SApos	30	6	36
	SAneg	1	68	69
	Total	31	74	105
**Agreement**		93%		
**Kappa coefficient**		0.85	(CI 0.74-0.96)	
A. Nasal samples. SApos: *S. aureus* positive; SAneg: *S. aureus* negative; CI: confidence interval.				
**Pharyngeal samples**		**Investigator-samples**		**Total**
		**SApos**	**SAneg**	
**Self-samples**	SApos	23	13	36
	SAneg	5	64	69
	Total	28	77	105
**Agreement**		83%		
**Kappa coefficient**		0.60	(CI 0.43-0.76)	

B. Pharyngeal samples. SApos: *S. aureus* positive; SAneg: *S. aureus* negative; CI: confidence interval.

Table 2 shows that, compared to the site-specific gold standard, nasal self-samples have a sensitivity of 97% (CI 86-100%), and pharyngeal self-samples have a sensitivity of 88% (CI 75-95%).

**Table 2:** Self-samples versus gold standard per sampling site

**Nasal samples**		**Gold standard**		**Total**
		**SApos**	**SAneg**	
**Self-samples**	SApos	36	0	36
	SAneg	1	68	69
	Total	37	68	105
**Sensitivity**		97%	(CI 86-100%)	
A. Nasal samples. SApos: *S. aureus* positive; SAneg: *S. aureus* negative; CI: confidence interval.				
**Pharyngeal samples**		**Gold standard**		**Total**
		**SApos**	**SAneg**	
**Self-samples**	SApos	36	0	36
	SAneg	5	64	69
	Total	41	64	105
**Sensitivity**		88%	(CI 75-95%)	

B. Pharyngeal samples. SApos: *S. aureus* positive; SAneg: *S. aureus* negative; CI: confidence interval.

## Discussion

This experimental study shows that self-samples and investigator-samples are very similar in testing the presence of *S. aureus* in nares and pharynxes. For nasal samples, agreement is 93% with a kappa coefficient of 0.85, indicating excellent agreement, for pharyngeal samples agreement is 83% and the kappa coefficient is 0.60, indicating good agreement. This means that self-samples are appropriate for detection of *S. aureus* and MRSA.

Furthermore, when looking at the discordant pairs, nasal self-samples tend to yield more *S. aureus* compared to investigator-samples. A rational explanation might be that swabbing is more thoroughly done by persons themselves compared to swabbing by investigators, who press less hard, resulting in fewer microorganisms picked up by the swab and a lower prevalence of *S. aureus*. For pharyngeal swabs the same can be stated, but results are less clear. This is probably due to the fact that taking swabs from tonsils or tonsillar arches is more complex, compared to swabs from the nares. In addition, the investigators observed that the instructions for pharyngeal samples were less well adhered to than the instructions for nasal samples.

This strengthens the conclusions from Lautenbach et al., although agreement percentages are slightly different (82% for nares and 91% for throat) [8]. Moreover, using the information given in this reference, kappa coefficients can be calculated, which are different from the coefficients found in this study (kappa coefficients of 0.28, CI −0.05-0.60 for nares and 0.80, CI 0.64-0.97 for throat). Possible explanations for these discrepancies can be the different populations studied (US inpatients versus healthy subjects from the Netherlands) or different methods used (strict or random sampling order, type of instructions). Hanselman et al. have used nasal self-sampling, and found *S. aureus* percentages in US teachers consistent with literature, indicating appropriateness of self-sampling [12]. More studies demonstrate the usefulness of self-samples, albeit from other anatomic sites and for other microorganisms, as human papilloma virus, group-B streptococci, respiratory viruses, and sexually transmitted diseases [7,13-15].

The validity of nasal and pharyngeal self-sampling cannot be established entirely correctly, as there is no true gold standard. However, when comparing to combined self-samples and investigator-samples per site, nasal self-samples have a sensitivity of 97% (CI 86-100%), and pharyngeal self-samples have a sensitivity of 88% (CI 75-95%). Lautenbach et al. have calculated sensitivities based upon a gold standard of combined samples from nares, throat, axillae, groin and perineum, and found sensitivities of 91% (CI 80-97%) for nasal self-samples, and 67% (CI 53-79%) for throat self-samples [8].

This study has three limitations. The nursing personnel members are expected to have more prior knowledge of sampling methods compared to the standard outhospital person. This might lead to an overestimation of the adequacy and validity of self-sampling. Moreover, false sampling was not checked for by, for example, placing the swab on a standard sheep blood agar, which detects whether any viable microorganisms are present on the swab. However, the investigators were standing next to the subject when sampling took place, making false sampling unlikely. In addition, *S. aureus* prevalence corresponds to literature, confirming adequate sampling [1]. Checking for false sampling with blood agar probably is a useful suggestion when using self-samples in a research situation, however. Lastly, three investigators were involved in taking the investigator-samples, which might lead to slightly different sampling techniques. As these investigators are all well trained on sample taking and sampling techniques were discussed beforehand, we believe the effect of this variation is negligible. Data on who took which samples was unfortunately not recorded, giving no opportunity to verify this statement.

## Conclusions

In conclusion, self-sampling the nares and pharynx for the presence of *S. aureus* has an excellent and good agreement with investigator-samples, respectively. Self-sampling saves both time and costs, and enhances response rate, which can be extremely useful in outhospital study situations of e.g. community-acquired or livestock-associated MRSA.

## Abbreviations

CI: Confidence interval; MRSA: Methicillin-resistant *Staphylococcus aureus*; SA: *Staphylococcus aureus*; *S. aureus*: *Staphylococcus aureus*; US: United States.

## Competing interests

The authors declare that they have no competing interests.

## Authors’ contributions

BvC designed the study, gathered and interpreted data, performed statistical analysis, and drafted the manuscript. MvR participated in the design of the study, gathered data and participated in drafting the manuscript. MF participated in gathering data. JK conceived the study, particpated in coordination an interpretation of data, and helped to draft the manuscript. All authors read and aproved the final manuscript.

## Authors’ information

BvC, MvR and JK conduct multiple outhospital studies for methicillin-resistant *S. aureus*, where self-sampling is very common.

## Supplementary Material

Additional file 1 **Instruction for *S. aureus *sampling.** Click here for file
