# Supplementary material for: Self-sampling is appropriate for detection of Staphylococcus aureus: a validation study
Source: Antimicrob Resist Infect Control. 2012 Nov 8;1:34. doi: 10.1186/2047-2994-1-34 (PMC3546066; doi:10.1186/2047-2994-1-34)
Supplement: Additional file 1 — Instruction for S. aureus sampling. [file 2047-2994-1-34-S1.doc]

#### Instruction for *S. aureus* sampling

***In case of S. aureus the following sites are sampled:***

Nose and Throat.

## Sampling method for S. aureus

Nose The inner side of both nostrils, especially in the tip of the nose. Swab in a turning movement with a sterile swab.


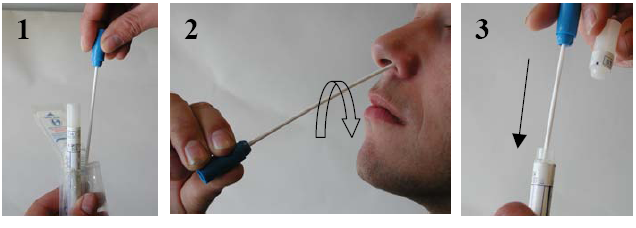


Throat Both tonsils or, if tonsils are removed, tonsillar arches. Swab in a turning movement with a sterile swab.


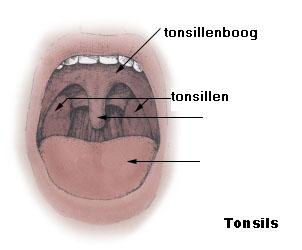


tonsillar arch

tonsils

Every sample is taken with a different swab.

If there are any questions, feel free to contact the Department of Infection Prevention, telephone xxx-xxxxxxx.
